# Life destiny of erythrocyte in high altitude erythrocytosis: mechanisms underlying the progression from physiological (moderate) to pathological (excessive) high-altitude erythrocytosis

**DOI:** 10.3389/fgene.2025.1528935

**Published:** 2025-04-02

**Authors:** Xiaoying Zhou, Quanwei Bao, Yu Cui, Xiaoxu Li, Chengzhong Yang, Yidong Yang, Yuqi Gao, Dewei Chen, Jian Huang

**Affiliations:** ^1^ Department of High Altitude Physiology and Pathology, College of High Altitude Military Medicine, Army Medical University, Chongqing, China; ^2^ Key Laboratory of Extreme Environmental Medicine, Ministry of Education, Chongqing, China; ^3^ Department of Emergency Medicine, Daping Hospital, Army Medical University, Chongqing, China; ^4^ College of High Altitude Military Medicine, Army Medical University, Chongqing, China

**Keywords:** high-altitude excessive polycythemia, chronic mountain sickness, excessive erythrocytosis, hypoxia, hypoxia induced factor

## Abstract

High-altitude polycythemia (HAPC) represents a pathological escalation of the physiological erythrocytosis induced by chronic hypoxia exposure. While moderate erythroid expansion enhances oxygen delivery, HAPC manifests as hematologic disorder characterized by hemoglobin thresholds (≥21 g/dL males; ≥19 g/dL females) and multi-organ complications including microcirculatory thrombosis, right ventricular hypertrophy, and uric acid dysmetabolism. This review critically evaluates the continuum between adaptive and maladaptive polycythemia through multiscale analysis of erythrocyte biology. We integrate genomic predisposition patterns, bone marrow erythroid kinetic studies, and peripheral erythrocyte pathophenotypes revealed by multi-omics profiling (iron-redox proteome, hypoxia-metabolome crosstalk). Current diagnostic limitations are highlighted, particularly the oversimplification of hemoglobin cutoffs that neglect transitional dynamics in erythrocyte turnover. By reconstructing the erythroid life cycle—from hypoxia-sensitive progenitor commitment to senescent cell clearance—we propose a phase transition model where cumulative epigenetic-metabolic derangements overcome homeostatic buffers, triggering pathological erythroid amplification. These insights reframe HAPC as a systems biology failure of erythroid adaptation, informing predictive biomarkers and targeted interventions to preserve hematological homeostasis in hypoxic environments.

## 1 Introduction

Increased red blood cell (RBC) production has been recognized as a hallmark of high-altitude acclimatization since Viault’s seminal 1980 observation. While moderate elevation of RBC count or hemoglobin mass ([Hb]) enhances oxygen transport capacity to counteract hypoxic stress, excessive erythrocytosis paradoxically compromises circulatory function. This review defines physiological high-altitude erythrocytosis as adaptive hemoglobin increases, whereas pathological high-altitude polycythemia (HAPC) refers to maladaptive overproduction that impairs capillary oxygen delivery (Frietsch et al., 2007), elevates blood viscosity (Tremblay et al., 2019; Raberin et al., 2022; Stauffer et al., 2020; Champigneulle et al., 2023), induces microcirculatory congestion (Mairbäurl, 2021), and exacerbates tissue hypoxia (Manohar et al., 1982).

HAPC also termed chronic mountain sickness (CMS) or Monge’s disease. It develops through prolonged (>2 years) hypoxic exposure and is formally defined by the International Society for Mountain Medicine as: hemoglobin ≥21 g/dL (male) or ≥19 g/dL (female) plus hypoxic symptoms (dyspnea, sleep disturbance, cyanosis, etc.) in high-altitude residents (>2500 m) (León-Velarde et al., 2005).

The Qinghai diagnostic system quantifies CMS severity using combined hemoglobin levels and symptom scores: total scores categorize cases as absent (0–5), mild (6–10), moderate (11–14), or severe (≥15) (León-Velarde et al., 2005). Notably, while excessive erythrocytosis (EE) is diagnostic prerequisite for diagnosing HAPC, but there are cases with high CMS scores without EE (Villafuerte et al., 2022; Champigneulle et al., 2022). This diagnostic framework raises two key questions: (1) whether current EE thresholds overlook subclinical hypoxia impacts, and (2) how non-erythrocytic mechanisms contribute to CMS pathogenesis independently of hemoglobin levels (Hancco et al., 2020b; Hancco et al., 2020a; Jiang et al., 2014a).

The central knowledge gap lies in distinguishing hypoxia-driven physiological erythropoiesis from pathological overproduction. Emerging evidence suggests this transition involves lifespan-regulated erythrocyte heterogeneity through three mechanisms: (i) genetic predisposition to impaired hypoxic sensing, (ii) dysregulated hematopoietic homeostasis, and (iii) adaptive failure in peripheral RBC phenotypes. By elucidating these molecular checkpoints, this review may help identify potential biomarker-defined thresholds for optimal erythrocytosis (sufficient for oxygen delivery yet avoiding hemodynamic overload), which could ultimately pave the way for precision management strategies in HAPC.

## 2 Diagnostic challenges in differentiating physiological vs. pathological polycythemia

**FIGURE 1:**
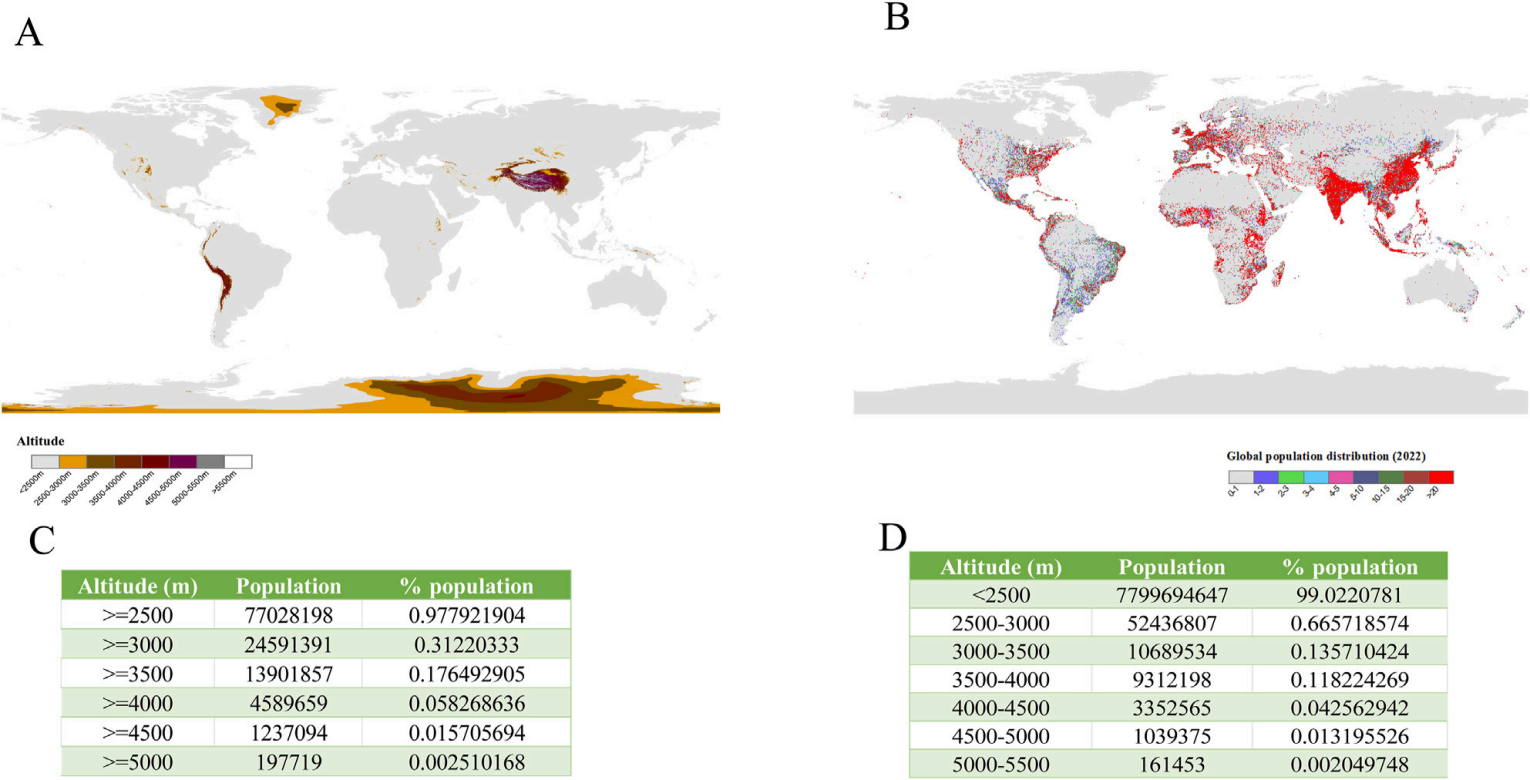
Global geospatial patterns of high-altitude regions and population distribution. **(A)** Global elevation distribution and **(B)** population density patterns were analyzed using ArcGIS 10.8 software (ESRI). Elevation data originated from the Global Multiresolution Terrain Elevation Data 2010 (GMTED2010; 30-arcsecond resolution, ∼1 km^2^ grid), with population statistics derived from the LandScan Global 2022 database. **(C)** Elevation zones were classified through DEM-based raster calculations, while **(D)** population distribution mapping employed conditional analysis with selection criteria.

Global high-altitude (≥2,500 m) populations reached 140 million in 1995 (Moore et al., 1998), fluctuating between 74.9 million (2017) and 81.6 million (2019) (Beall, 2014; Romeo et al., 2020; Tremblay and Ainslie, 2021), with our 2022 analysis indicating a decline to 77 million (0.98% of global population) (Figure 1). CMS affects 5%–31% of this population (Sims et al., 2023), with prevalence variations reflecting three determinants: (1) altitude gradients, (2) ethnic adaptation histories, and (3) individual acclimatization capacity.

Comparative analysis of four long-term adapted populations reveals distinct hematological adaptations (Villafuerte and Corante, 2016; Beall et al., 2002). Tibetans (Tibetan plateau, ∼30,000 years adaptation) and Sherpas (Himalayas, ∼12,000 years) exhibit lower hemoglobin concentrations ([Hb] = 14–16 g/dL). Aymarans (Andes, ∼5,000 years adaptation) maintain higher baseline [Hb] (17–19 g/dL) [31] with elevated CMS susceptibility. Amharas (Ethiopia, adaptation timeline undetermined) demonstrate normal sea-level [Hb] values despite chronic hypoxia. Pre-2004 epidemiological data showed exponential CMS increases with altitude on the Tibetan Plateau: 1.05% at 2,261–2,980 m, 3.75% at 3,128–3,980 m, and 11.83% at 4,000–5,226 m (peaking at 33% in ≥60-year-olds. The presence or absence of heart failure was not indicated in this article) (Monge et al., 1989). Post-Qinghai Consensus studies report: Global CMS estimates as 5%–10%. Subsequent studies indicated a CMS prevalence of 28.7% at 3,000–4,200 m in North India’s Himalayas, contrasting with 13.73% at 3,000–4,150 m in another study (Negi et al., 2013; Sahota and Panwar, 2013) (Table 1).

**TABLE 1:** The prevalence of CMS and EE.

Region	Address	Altitude (m)	Residence time	Age (years)	Qinghai CMS	EE without CMS	Year	References
Global	Global	>2,500	NA	NA	5%–10%	NA	2005	León-Velarde et al. (2005), Monge et al., (1989)
East Africa	Amharas Region	3,530	Native residents	14–86	0	0	2002	Beall et al. (2002)
Qinghai-Tibet Plateau	Qinghai, Xinjiang	3,175–5,380	Male Chinese highland > 5 years	NA	1.25%–36.58%	NA	2012	Li et al. (2012)
	Spiti Valley in the northern state of Indian Himalayas	3,000–4,200	Natives	≥20	28.7%	NA	2013	Negi et al. (2013)
	Sirmaur, Kinnaur and Lahaul and Spiti in Himachal Pradesh, India	2,350–4,150	Most of them > 5 years	17–75	Mean 6.17% over 3,000 m 13.73%	NA	2013	Sahota and Panwar (2013)
	Qinghai-Tibetan plateau	3,700–5,000	Han males for 2–96 months	24.08 ± 5.66 years	17.8%	NA	2014	Jiang et al. (2014a)
	Tibetan Plateau	3,600–3,700	Immigrants > 2 years	15–45 years	25.8% (28.7% in males and 9.4% in females	NA	2021	Song et al. (2023)
Andes Mountains	La Rinconada, south of Peru	5,100–5,300	Residents	29 [24–41]	31%	76%	2005	León-Velarde et al. (2005)
	Carhuamayo and Junin in Andes	>3,000	>30 years	35–75	32.6%	9.7%	2010	Gonzales et al. (2013)
	Puno (Department in southwestern Peru.)	>3,825	Most Aymaran	≥35	6%	4.5%	2014	De Ferrari et al. (2014)
	La Rinconada, south of Peru	5,100–5,300	Permanent residents	18–57	14%	44%	2017	Hancco et al. (2020a)
	La Rinconada, south of Peru	5,100–5,300	at least 1 year of residency in La Rinconada	From 29 [24–41] to the following 14 years	4.4 cases by person-years	6.3 cases by person-years	2005–2019	Champigneulle et al. (2022)

Above all, three fundamental limitations undermine current diagnostic thresholds (León-Velarde et al., 1993; Monge-C et al., 1992; Monge et al., 1989). i) Historical basis: EE criteria derived from 1990s Peruvian data at 4,340 m (defined as [Hb] > 2SD above local mean). ii) Altitude-dependent validity: Fixed thresholds underestimate EE prevalence below 3,000 m (physiological [Hb] suppression) yet overestimate above 5,000 m (La Rinconada data: CMS 14%–31% vs. EE 44%–76% (De Ferrari et al., 2014; Hancco et al., 2020a). iii) Ethnic hematological variance: Native highlanders (Tibetans/Aymarans) maintain lower [Hb] (14–19 g/dL) than immigrant populations (Jiang et al., 2014a; Beall, 2007; Beall et al., 1998). Longitudinal cohort data indicate rising incidence rates of 4.4 CMS and 6.3 EE cases per person-year over 14 years (León-Velarde et al., 2005; Champigneulle et al., 2022; Oberholzer et al., 2020). We consequently advocate for diagnostic frameworks incorporating altitude-adjusted thresholds and ethnic-specific [Hb] baselines to improve clinical distinction between adaptive and pathological erythrocytosis.

## 3 Genetic susceptibility in HAPC: population-specific adapt mechanisms

The transition from physiological erythrocytosis to pathological CMS is governed by genetic susceptibility under hypoxic stress. Indigenous populations such as Tibetans and Andeans exhibit distinct evolutionary adaptations to hypoxia, while lowlanders remain vulnerable to CMS. This part synthesizes evidence from genetic, epigenetic, and functional studies to elucidate how population-specific variants in hypoxia-related genes modulate erythropoietic responses, offering insights into the molecular basis of adaptive and maladaptive polycythemia.

### 3.1 Tibetans

Tibetans exhibit unique hypoxia adaptation mechanisms, including an enhanced hypoxic ventilatory response (HVR) and improved oxygen diffusion efficiency rather than relying on elevated [Hb] levels. Genome-wide analyses have identified *EPAS1* (encoding HIF-2α) as a key gene under positive selection, with a 78% allele frequency difference between Tibetans and Han Chinese (Jeong et al., 2014). Subsequent studies confirmed its role in maintaining low [Hb] via the HIF-EPO pathway, preventing CMS (Simonson et al., 2010; Yi et al., 2010; Song et al., 2020; Shi et al., 2023; Ge et al., 2023; Storz and Cheviron, 2021; Beall et al., 2010). Notably, the *EPAS1* rs149594770 allele (57.2% prevalence in Tibetans) reduces promoter activity and suppresses hypoxia-responsive erythropoiesis (Peng et al., 2017; Deng et al., 2019).

*EGLN1* (encoding PHD2) and *PPARA* further contribute to this adaptation. Each variant allele at *EGLN1* or *PPARA* loci decreases [Hb] by ∼1.7 g/dL (Peng et al., 2011; Simonson et al., 2010; Semenza, 2020). Functional studies reveal Tibetan-specific *EGLN1* mutations (c.12C > G [85%] and c.380G > C [20%]) alter PHD2 kinetics: the p. [Asp4Glu; Cys127Ser] double mutation increases HIF-2α hydroxylation efficiency (Km for O_2_ reduced by 30%) while disrupting interaction with ribosomal chaperone NACA due to a Pro-Xaa-Leu-Glu (PXLE) motif alteration (Lorenzo et al., 2014; Tashi et al., 2017; Yang et al., 2017; Lorenzo et al., 2014). This dual mechanism enhances HIF-2α degradation in renal tissues but preserves HIF-2α activity in the carotid body, augmenting HVR (Song et al., 2022).

PPARA downregulation shifts fatty acid metabolism from β-oxidation to ω-oxidation, reducing oxygen demand and oxidative stress (Song et al., 2020; Horscroft et al., 2017; Ge et al., 2023). Concurrently, the *HMOX2* rs4786504 C allele enhances heme catabolism, further lowering [Hb] (Yang et al., 2016). Additional genotype-phenotype associations have been reported (Guo et al., 2020).

Despite these adaptations, Tibetans at extreme altitudes (>4,500 m) may develop CMS due to: 1) Environmental triggers: Dietary factors (Zhang et al., 2017; Wang et al., 2022b; Cui et al., 2022) and nocturnal hypoxemia (Rexhaj et al., 2016; Julian et al., 2013). 2) Genetic susceptibility: Gain-of-function *EPAS1* variants (e.g., rs13419896) (Xu et al., 2015a), *PIK3CD/COL4A3* SNPs (Fan et al., 2018; Zhao et al., 2017), and *CYP17A1/CYP2E1* polymorphisms (Xu et al., 2015b). 3) Epigenetic dysregulation: Aberrant methylation of *ABCA1*, *TGF-β*, and *BMPR2* (Zhaxi et al., 2024).

While *EPAS1/EGLN1/PPARA* selection blunts EPO-driven erythropoiesis, alternative mechanisms (e.g., higher plasma volume vs. Andeans) may also contribute to low [Hb] (Hansen et al., 2021; Stembridge et al., 2019). However, extreme hypoxia or deleterious variants can override these protections, highlighting limitations of genetic adaptation under severe hypoxic stress.

### 3.2 Andeans

The Aymaras, another indigenous population adapted to high-altitude environments, demonstrate elevated hemoglobin levels as an evolutionary response to hypoxia. While Andeans generally show evidence of positive genetic selection, they exhibit higher susceptibility to CMS than Tibetans, though less than lowland populations. Notably, no canonical genetic variants directly drive high [Hb] in Andeans. Instead, indirect genetic modifiers influence CMS risk by regulating secondary factors. For instance, Andeans with lower [Hb] and improved oxygen saturation phenotypes carry adaptive variants such as *EPAS1* (rs570553380) and *EGLN1* (Brutsaert et al., 2019), which reduce HIF target gene expression by modulating HIF-2α structure, stability, and transcriptional activity (Lawrence et al., 2024; Eichstaedt et al., 2017). The H194R mutation in HIF-2α further protects against hypoxia-induced pulmonary hypertension by suppressing endothelin-1 transcription under low-oxygen conditions (Jorgensen et al., 2023).

Sentrin-specific Protease 1 (*SENP1*) and *ANP32D* have emerged as promising therapeutic targets for CMS following experimental validation (Zhou et al., 2013; Cole et al., 2014; Hsieh et al., 2016). For example, *FAM213A* and *SFTPD* variants linked to antioxidant activity (Valverde et al., 2015), and positively selected genes like *SP100*, *DUOX2*, and *CLC*—implicated in broad adaptive pathways (Jacovas et al., 2018), highlight the complexity of altitude adaptation. Additionally, elevated allele frequencies of cardiovascular-associated loci (rs11578671_*BRINP3*, rs34913965_*NOS2*, rs10744822_*SH2B1*, and *TBX5*) in Aymaras suggest these variants may indirectly support elevated [Hb] while mitigating hypoxic damage or EE complications (Crawford et al., 2017). This genetic interplay partially explains why some Andeans develop EE without progressing to CMS.

Epigenetic mechanisms complement genetic adaptations in environmental acclimatization. Bigham’s work revealed an inverse correlation between years of high-altitude residency and EPAS1 methylation, coupled with increased LINE-1 methylation (Childebayeva et al., 2019). Collectively, these findings illustrate that Andeans harbor both EE susceptibility alleles and cardioprotective variants that counteract high circulatory pressure. While their clinical presentations often remain subpathological (CMS scores < 6), latent risks persist. Although this study classifies their condition as pathological erythrocytosis, further investigation is warranted to confirm this categorization.

### 3.3 Lowlanders

In Han Chinese populations, specific genetic variants—rs8065364 (*CARD14*), rs3025033 (*VEGFA*), and rs726354 (*SENP1*)—may contribute to HAPC pathogenesis (Chen et al., 2016). The mtDNA 10609 variant has also been linked to HAPC via hypoxia-induced ROS elevation (Jiang et al., 2014b), while *LRRC18 and HCAR3* are predicted as hub genes in this condition (Wang et al., 2022a). However, the precise molecular mechanisms, functional roles, and interactive networks of these candidates remain poorly characterized. Future studies must clarify their contributions to HAPC development, including detailed explorations of their downstream targets and pathway interactions.

**FIGURE 2:**
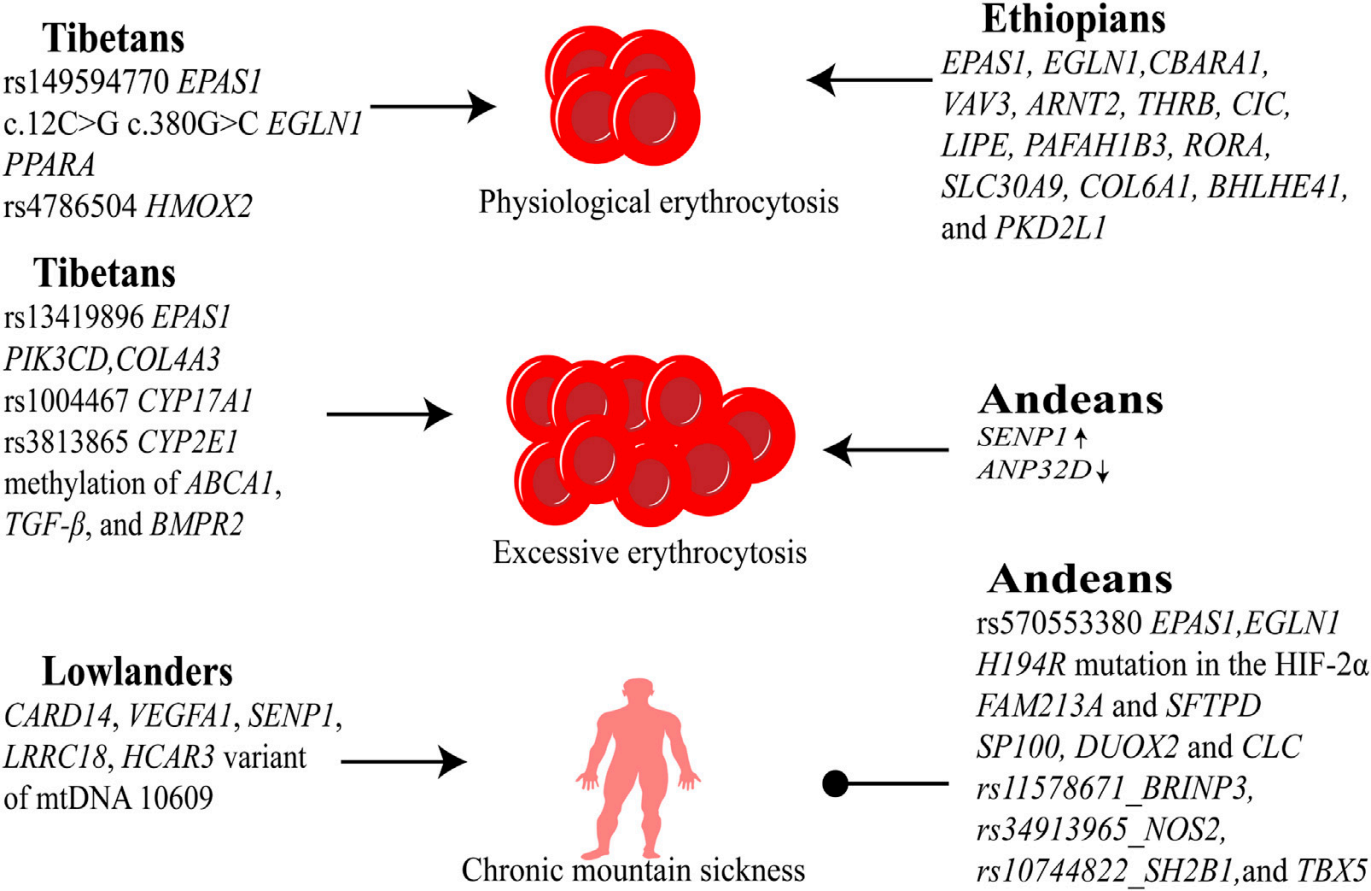
Genetic adaptations to high-altitude hypoxia. Tibetans carry protective mutations in *EPAS1* (HIF-2α regulation), *EGLN1* (HIF-1α degradation), and *PPARA* (fatty acid metabolism), mitigating EE; however, susceptibility polymorphisms (e.g., *CYP17A1*) emerge at extreme altitudes, increasing CMS risk. Andeans exhibit high EE prevalence linked to SENP1-enhanced HIF-1α stability but retain cardiovascular-protective alleles (e.g., *NOS2*), reducing CMS incidence despite erythrocyte overproduction. Ethiopians maintain near-sea-level blood oxygen saturation via distinct genetic selection (e.g., *BHLHE41*), avoiding EE/CMS through oxygen efficiency optimization rather than erythrocyte modulation.

Ethiopian highlanders exhibit no clinical manifestations of EE or CMS. Tibetan populations possess genetic variants conferring relative resistance to these conditions, though migration to higher altitudes reveals unique susceptibility alleles. Andeans display well-defined EE risk genes alongside cardioprotective variants that ameliorate vascular damage. In contrast, lowland populations demonstrate heightened susceptibility to CMS and EE but lack strong genetic predispositions (Figure 2).

## 4 Hematopoietic homeostasis dysregulation: a central mechanism in the transition from physiological to pathological erythrocytosis

### 4.1 Cumulative effects of EPO signaling

The HIF-EPO axis, a critical oxygen-sensing mechanism, enhances oxygen delivery by stimulating hemoglobin production during hypoxemia. EPO drives erythropoiesis through two complementary pathways: 1) direct stimulation of erythroid precursor proliferation via receptor binding, and 2) hematopoietic stem/progenitor cell (HSPC) reprogramming that preferentially commits multipotent progenitors (MPP1/MPP2) to erythroid lineage through transcriptional regulation. Chronic EPO exposure induces a paradigm shift in hematopoiesis—transgenic models demonstrate hematopoietic stem cell (HSC)-directed erythropoiesis that bypasses conventional MPP-mediated differentiation hierarchies through epigenetic remodeling (Eisele et al., 2022; Grover et al., 2014).

However, the erythropoietic efficacy of chronic EPO elevation remains debated. Cross-population analyses reveal striking geographical variations in baseline EPO levels: 1) Chilean highlanders: 9.6 ± 4.3 mIU/mL (non-CMS) vs. 45 mIU/mL (CMS); 2) Hypoxia-adapted Sherpas: 8.4 ± 7.1 mIU/mL. 3) Northern Indian highlanders: 2.13–2.18 IU/L (sex-independent). 4) Andean CMS patients: 21.6 ± 6.7 pg/dL vs. 12.3 ± 2.9 pg/dL (healthy controls) (Winslow et al., 1989; Yanamandra et al., 2019; Bermudez et al., 2020; Robach et al., 2004). These findings suggest persistent hypoxic signaling in high-altitude populations, yet the “cumulative EPO” hypothesis for pathological erythropoiesis in CMS faces clinical contradictions. While CMS cohorts show marginally elevated EPO compared to non-EE controls, experimental models reveal decoupled EPO-erythrocyte dynamics—transient EPO spikes during intermittent hypoxia fail to induce proportional erythroid responses. Notably, high-altitude adaptation follows a distinct temporal pattern: EPO surges 24–48 h post-ascent, declines gradually, and stabilizes near sea-level concentrations within weeks, suggesting complex feedback regulation beyond simple hypoxic stimulation (Painschab et al., 2015; Gore et al., 2006; Dainiak et al., 1989; Faura et al., 1969).

Emerging evidence reveals an explanation in cumulative effects of EPO signaling. While systemic EPO levels remain comparable between CMS and non-CMS individuals, localized EPO signaling may drive pathological erythropoiesis. Approximately 90% of EPO is synthesized by the kidneys, however, extrarenal organs and tissues also contribute to EPO production, which may be involved in the symptoms of CMS other than hemoglobin. Specifically, erythroid progenitor cells, osteoblasts, and CD169^+^ macrophages within the bone marrow microenvironment have been identified as functional EPO-secreting cells. These marrow-derived cells establish an autocrine/paracrine regulatory circuit, where locally produced EPO directly modulates erythroid differentiation. This compartmentalized signaling could explain individual CMS susceptibility variations despite similar systemic EPO levels, suggesting bone marrow microenvironments differentially amplify hypoxic signals through tissue-specific EPO production (Chow et al., 2013; Su et al., 2015).

Another explain is soluble erythropoietin receptor (sEPOR), an antagonist of EPO, which blocks EPO and reduces its availability (Villafuerte, 2015). Despite comparable EPOlevels, a reduction in sEPOR concentrations elevates the EPO/sEPOR ratio, thereby amplifying EPO-mediated erythroid proliferative signaling. Andean populations suggest that an increase in circulating erythropoietin receptor (Epo-R) during gestation in these populations may be an important vascular adaptation factor to achieve a successful pregnancy in the face of high-altitude hypoxia (Wolfson et al., 2017; Villafuerte et al., 2014). This finding suggested that the ratio of EPO to sEPOR might be associated with CMS (Vizcardo-Galindo et al., 2020). This finding suggested that EPO/sEPOR ratio might be associated with CMS (Villafuerte et al., 2014; Vizcardo-Galindo et al., 2020; Wolfson et al., 2017).

The relationship between hypoxemia, erythropoietin (EPO), and EE reveals unexpected phenotypic heterogeneity in high-altitude populations. Approximately 27% of highlanders maintaining SpO_2_ levels above 83% develop EE, while conversely, 28% of individuals with severe hypoxemia (SpO_2_ < 83%) exhibit [Hb] within altitude-adjusted normative ranges. Notably, 47% of non-EE individuals demonstrate elevated serum EPO levels, collectively indicating that neither profound hypoxemia nor sustained EPO elevation constitutes an absolute prerequisite for EE development (Villafuerte et al., 2022). This discordance implies additional pathophysiological mechanisms driving erythroid overproduction.

### 4.2 Erythroid hyperproliferation and suppression of ineffective hematopoiesis

The investigation of HAPC hinges on unraveling the interplay between hypoxia-driven erythropoietic adaptation and its pathological deviations. Animal models simulating hypobaric hypoxia—defined by EE thresholds analogous to human clinical criteria (Hb ≥ 21 g/dL in males, Hb ≥ 19 g/dL in females)—have provided critical insights into bone marrow reprogramming under chronic hypoxia. These studies revealed a coordinated shift in hematopoietic dynamics, marked by selective expansion of megakaryocyte-erythrocyte progenitors (MEP) at the expense of granulocyte-macrophage progenitors (GMP). This lineage bias correlates with transcriptional reprogramming, characterized by sustained GATA-1 upregulation and concurrent suppression of PU.1. Further mechanistic exploration identified epigenetic alterations, particularly VHL promoter hypermethylation in bone marrow cells, which stabilizes HIF-2α by impeding its ubiquitination-dependent degradation, thereby amplifying hypoxic signaling cascades (Li et al., 2011; Yang et al., 2021).

While these models have elucidated key aspects of erythroid hyperproliferation, their translational relevance remains tempered by interspecies physiological disparities. Rodents inherently exhibit higher baseline hemoglobin levels (∼15 g/dL) compared to humans (∼13–14 g/dL), raising questions about the clinical equivalence of EE thresholds. Moreover, the reductionist nature of current models—focusing predominantly on hematological parameters—overlooks the multisystemic manifestations of CMS, which encompasses clinical syndromes (Li et al., 2011; Chen and Kaul, 1994). This discrepancy underscores the necessity of contextualizing experimental findings within the broader pathophysiological framework of CMS. Crucially, while hemoglobin concentration serves as a cardinal biomarker, its elevation reflects neither the totality of disease mechanisms nor the clinical heterogeneity observed in high-altitude populations. Thus, conclusions derived from these models demand cautious interpretation, emphasizing the need for integrative approaches that bridge molecular insights with systemic pathophysiology.

Researchers have utilized human bone marrow samples to investigate the mechanisms of HAPC. *In vitro* cultures of bone marrow mononuclear cells from individuals with CMS and non-CMS revealed elevated levels of CD71^+^ cells, polychromatophilic normoblasts, and orthochromatic normoblasts in the CMS group. This phenomenon was attributed to reduced erythroblast apoptosis, driven by higher mitochondrial membrane potential (MMP), increased Bcl2 expression, and decreased Bax levels (Ma et al., 2019). The anti-apoptotic effect in CMS was further linked to enhanced activation of the PI3K-Akt signaling pathway in response to EPO (Harada et al., 2015). “Ineffective erythropoiesis” was defined as mature erythrocytes partially declining with erythroid proliferation. In physiological conditions, ineffective erythropoiesis maintained a certain level, but in HAPC, it decreased significantly, reflecting reduced erythroblast apoptosis (Wang et al., 2018). EPO has been identified as a key driver of this phenotypic shift, reinforcing the hypothesis that pathological erythrocytosis arises from the superimposed effects of EPO signaling.

The investigation of HAPC faces inherent challenges in animal models, where interspecies physiological disparities—particularly rodents’ elevated baseline erythrocyte counts and superior hypoxic adaptation—limit their capacity to distinguish pathological versus physiological responses. These constraints have driven researchers toward human-centric approaches, though bone marrow sampling remains ethically and logistically challenging. Pioneering work by Gabriel G. Haddad circumvented this barrier by reprogramming peripheral blood mononuclear cells (PBMCs) from chronic mountain sickness (CMS) patients and controls into induced pluripotent stem cell (iPSC)-derived CD34^+^ hematopoietic progenitors (Azad et al., 2016). This innovative model successfully recapitulated the EE phenotype under hypoxic conditions, enabling mechanistic dissection of CMS pathophysiology.

Critical insights emerged from comparative analyses of these cellular models: the SUMO-specific protease SENP1, identified as an adaptive genetic factor in Andean highlanders, regulates erythroid expansion through coordinated post-translational modifications. In non-CMS progenitors, hypoxia-triggered SENP1 mediates sumoylation-dependent suppression of GATA1 and HIF-1α, subsequently downregulating the anti-apoptotic factor Bcl-xL to constrain erythroid output. CMS-derived cells, however, exhibit marked SENP1 deficiency, disrupting this regulatory circuit and permitting uncontrolled erythroblast proliferation via sustained Bcl-xL activity. This mechanistic divergence underscores how genetic variation modulates hypoxic adaptation thresholds, transforming compensatory erythropoiesis into pathological erythrocytosis (Azad et al., 2016; Bermudez et al., 2020). Comparative genomic analyses have revealed a critical distinction between CMS and non-CMS through the identification of differential SNPs within the AT-rich interaction domain 1B [*ARID1B* (Zhou et al., 2013)]. ARID1B demonstrates dual regulatory patterns under hypoxic conditions: direct transcriptional activation by HIFs and secondary induction through reactive oxygen species (ROS)-mediated signaling pathways. Mechanistic studies demonstrate that ARID1B exerts precise epigenetic control over erythroid maturation through two complementary pathways. First, it modifies chromatin architecture at the GATA1 locus to suppress this critical erythroid transcription factor. Second, it simultaneously enhances chromatin accessibility at the p53 tumor suppressor locus. This coordinated regulation creates a molecular rheostat that reduces erythroid progenitor proliferation while increasing apoptotic susceptibility. Notably, CMS patients exhibit significantly lower ARID1B expression levels compared to altitude-adapted controls. The impaired ARID1B expression observed in CMS patients results in dysregulated erythroid cell dynamics, ultimately contributing to pathological polycythemia (Azad et al., 2022). The ARID1B-mediated pathway represents a crucial checkpoint that becomes compromised in CMS pathogenesis, highlighting its potential as both a diagnostic biomarker and therapeutic target.

Transcriptomic profiling of PBMC-derived native CD34^+^ hematopoietic progenitor cells has uncovered a critical role for long noncoding RNAs (lncRNAs) in CMS pathogenesis. A prominent finding is the marked upregulation of *hypoxia-induced kinase-mediated erythropoietic regulator* (*HIKER, LINC02228*) in CMS patients. Functional studies reveal that HIKER amplifies erythroid progenitor expansion by stabilizing the regulatory β-subunit of casein kinase 2 (CSNK2B)—a kinase with evolutionarily conserved roles in erythropoiesis—thereby enhancing the expression of GATA1. This dysregulated HIKER-CSNK2B-GATA1 axis drives pathological erythroid hyperplasia, as evidenced by increased BFU-E colony formation in CMS-derived progenitors (Azad et al., 2023).

Intriguingly, sexual dimorphism in CMS susceptibility emerges from hormone-dependent modulation of GATA1 pathway. *In vitro* models demonstrate that estrogen suppresses erythroid proliferation through coordinated downregulation of GATA1, heme biosynthesis enzyme ALAS2, and anti-apoptotic factor Bcl-xL (Azad et al., 2021). Conversely, testosterone exhibits a dose-dependent association with CMS severity in males, potentially exacerbating erythrocytosis through androgen receptor-mediated transcriptional programs (Gonzales et al., 2009).

**FIGURE 3:**
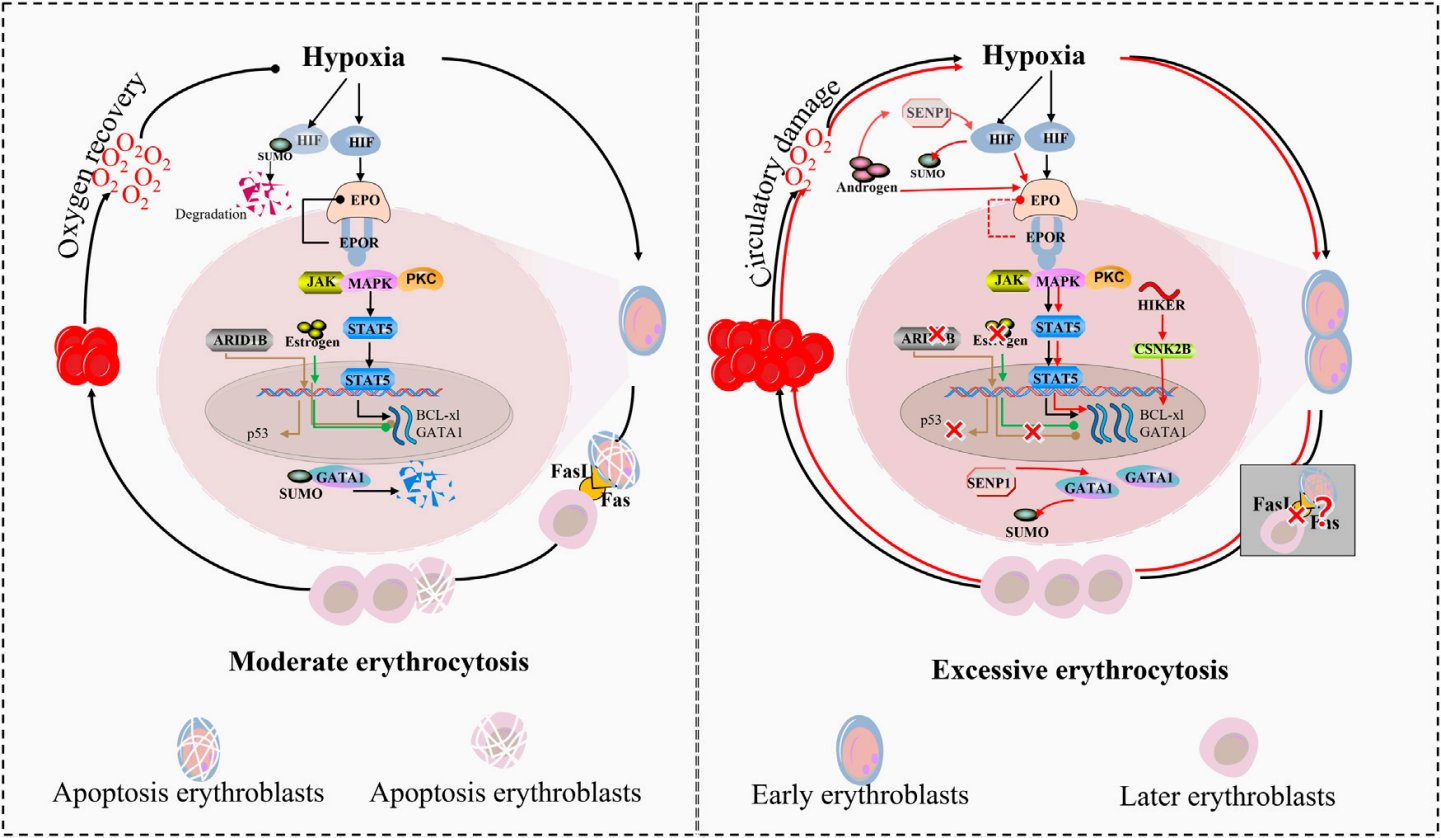
Divergent regulatory pathways in moderate erythropoiesis and excessive erythrocytosis. The regulation of erythropoiesis under hypoxia involves a tightly balanced molecular network that diverges dramatically between physiological adaptation and pathological erythrocytosis in CMS. Central to this dichotomy is the HIF signaling axis, which interfaces with chromatin remodeling, noncoding RNA networks, and hormone signaling to control erythroid proliferation and apoptosis. 1) Hypoxic Sensing and Transcriptional Activation. Under physiological hypoxia, HIF-α stabilization initiates a controlled erythropoietic response through two counterbalanced mechanisms: HIF-α recruits SENP1 to deSUMOylate and stabilize GATA1; 2) Homeostatic restraint: Concurrently, ARID1B modulates chromatin accessibility at the GATA1 locus to limit transcriptional hyperactivity, and estrogen signaling suppresses ALAS2 via ERα-mediated repression. 3) Epigenetic-Noncoding RNA Crosstalk. The long noncoding RNA HIKER (LINC02228) serves as a pivotal rheostat: In physiological states, baseline HIKER expression supports CSNK2B-mediated GATA1 phosphorylation, facilitating normal erythroid maturation while maintaining sensitivity to ARID1B-mediated chromatin repression. In CMS, hypoxia-driven HIKER overexpression hyperactivates CSNK2B, rendering GATA1 resistant to ARID1B suppression. This epigenetic bypass results in sustained ALAS2 upregulation and heme overproduction. 4) Apoptosis-Proliferation Coupling. Late-stage erythroblast-derived FasL enforces population control through paracrine apoptosis of early progenitors: Physiologically, ARID1B-maintained p53 expression, creating negative feedback proportional to erythroblast density. In CMS, ARID1B deficiency reduces p53 and upregulates Bcl-xL, collectively blunting FasL-mediated apoptosis. This dual defect permits uncontrolled expansion of FasL-resistant progenitors. 5) Sexual dimorphism in CMS susceptibility emerges from hormone-pathway interactions: Estrogen reinforces physiological restraint by suppressing SENP1-HIF-α crosstalk. Testosterone exacerbates CMS by potentiating both AR-mediated proliferation, while inhibiting estrogen receptor signaling.

Under physiological conditions, the apoptosis-inducing ligand FasL is selectively expressed in late-stage erythroblasts, where it mediates apoptosis signaling to eliminate excess early-stage erythroblasts. This cell-autonomous feedback mechanism ensures homeostatic control of erythropoiesis by dynamically balancing progenitor proliferation and elimination, thereby preventing pathological erythroid expansion (Liu et al., 2006). In CMS, this critical regulatory axis appears to be dysregulated. While direct evidence of FasL pathway suppression remains unreported in CMS literature, clinical observations consistently demonstrate significantly reduced erythroblast apoptosis rates in CMS. The combined effects of epigenetic dysregulation (via ARID1B) and impaired paracrine apoptosis (via FasL) may synergistically drive uncontrolled erythroid expansion (Figure 3).

### 4.3 Pathological remodeling of the bone marrow microenvironment

The hematopoietic niche comprises stromal cells and a specialized extracellular matrix (ECM) enriched with cytokines (e.g., SCF, IL-3, IL-6), structural proteins (e.g., fibronectin, laminins), and adhesion molecules (e.g., VCAM-1, integrins). This microenvironment orchestrates hematopoiesis through three synergistic mechanisms: spatial regulation, metabolic support and cytokine crosstalk.

#### 4.3.1 Erythroblast Islands (EBI)

First described by Bessis (1958), EBIs are bone marrow-resident units central to coordinated erythropoiesis (Țichil et al., 2024). At the structural core of each EBI resides a central macrophage, which serves as both a physical scaffold and a functional regulator. This macrophage fulfills three critical roles: (1) phagocytic clearance of expelled nuclei during erythroblast enucleation, (2) membrane remodeling to facilitate reticulocyte maturation, and (3) metabolic support through iron recycling and trophic factor secretion (Țichil et al., 2024; Yeo et al., 2019).

Experimental evidence underscores the indispensability of EBI macrophages under hematopoietic stress. Selective macrophage depletion in murine models abolishes compensatory stress erythrocytosis while paradoxically ameliorating polycythemia vera manifestations, suggesting dual regulatory roles in physiological and pathological contexts (Li et al., 2020; Chow et al., 2013). Conversely, macrophage supplementation enhances erythrocyte production efficiency, highlighting their functional plasticity. Recent studies reveal a novel mitochondrial crosstalk mechanism: mature erythroblasts transfer mitochondria to macrophages via CD47-mediated signaling, reprogramming macrophage bioenergetics to sustain EBI homeostasis (Yang et al., 2022).

The EBI microenvironment demonstrates remarkable oxygen responsiveness. Dynamic interactions between macrophage α4/β1 integrins and erythroblast ICAM-4/VCAM-1 create oxygen-regulated adhesion complexes that modulate erythroblast retention/release. Hypoxia upregulates BMP4 and SCF expression in bone marrow stromal cells, with BMP4 levels increasing in CMS. These cytokines synergistically inhibit erythroid apoptosis while promoting differentiation (Harada et al., 2015).

The hematopoietic niche integrates immune regulation through specialized Th2 cell populations. Under hypobaric hypoxia, murine models demonstrate Th2 cell migration to bone marrow spaces with increasing of activin A and IL-9, amplifying erythroid progenitor expansion (Li et al., 2014). Synergistic action with macrophage-derived IL-3/IL-6 to bias HSC toward erythroid lineage commitment in HAPC (Li et al., 2011). The EBI ecosystem exhibits remarkable adaptive plasticity. While macrophage depletion strategies show therapeutic potential for polycythemia vera. The discovery of mitochondrial transfer mechanisms and oxygen-sensitive cytokine axes provides novel targets for managing CMS.

#### 4.3.2 Iron metabolism

Physiological iron metabolism initiates at intestinal epithelia where dietary ferrous iron (Fe^2+^) is absorbed via divalent metal transporter 1 (DMT1). Intracellular iron is either stored as ferritin (FT) nanocages or exported to circulation through ferroportin (FPN), the sole known iron efflux channel. Prior to entering plasma, exported Fe^2+^ undergoes oxidation to ferric state (Fe^3+^) by membrane-bound hephaestin, enabling binding to transferrin (Tf) for systemic distribution (Shah and Xie, 2014). The progress is governed by a multi-layered regulatory network. BMP ligands engage BMPR receptors, triggering phosphorylation of R-SMAD which complexes with CO-SMAD4 to activate HAMP transcription. Additionally, STAT3, HNF4α, and C/EBPα functionally synergize with SMAD complexes to amplify hepcidin expression. HIFs directly repress both BMP-SMAD signaling and co-regulators, establishing oxygen tension as a master switch for systemic iron availability. Hypoxia-driven erythropoiesis activates two interdependent regulatory loops. Firstly, EPO stimulates erythroblast secretion of erythroferrone (ERFE), which competitively inhibits BMP-SMAD signaling through TWSG1-mediated BMP ligand sequestration. Secondly, differentiation-associated factors GDF15 and TWSG1 cooperatively suppress SMAD phosphorylation efficiency, creating positive feedback to maintain iron mobilization for hemoglobin synthesis (Shah and Xie, 2014).

In healthy individuals, transferrin (Tf) maintains a saturation level of approximately 30%, with 70%–80% of systemic iron being allocated for heme biosynthesis. The canonical iron delivery pathway involves diferric Tf binding to transferrin receptor 1 (TfR1) on erythroid cell membranes, followed by receptor-mediated endocytosis and subsequent iron release within acidic endosomal compartments. In contrast to TfR1’s primary role in cellular iron acquisition, its homolog TfR2 has evolved distinct regulatory functions, predominantly serving as a sensor of circulating Tf saturation status rather than participating directly in iron transport. Under iron repletion (high Tf saturation), TfR2 interacts with EPOR complex, exerting inhibitory effects that attenuate EPO signal transduction during steady-state erythropoiesis. This regulatory mechanism ensures appropriate coordination between iron availability and RBC production. Conversely, during iron-deficient states (low Tf saturation), diminished Tf-TfR2 interactions relieve this inhibitory effect, thereby potentiating EPO receptor signaling. This adaptive response enhances stress erythropoiesis under conditions of hematopoietic challenge, demonstrating a sophisticated feedback system that prioritizes erythroid demands during systemic iron scarcity.

The regulation of iron metabolism under hypoxic conditions operates through tightly coordinated molecular mechanisms rather than indiscriminate iron mobilization. Central to this process are iron regulatory proteins 1 and 2 (IRP1/IRP2), which maintain systemic iron balance by post-transcriptionally modulating the expression of key iron-associated proteins. These RNA-binding proteins recognize conserved iron-responsive elements (IREs)—cis-acting hairpin structures in the untranslated regions of target mRNAs—to regulate proteins including ferritin (FT, iron storage), ferroportin (FPN, iron export), transferrin receptor (TfR, iron uptake), and δ-aminolevulinic acid synthase 2 (ALAS2, heme biosynthesis). Notably, hypoxia-induced erythrocytosis and iron acquisition are governed by the dynamic interplay between hypoxia-inducible factor 2α (HIF-2α) and IRP-mediated regulatory networks. Under iron-deficient conditions, Apo-IRP1 adopts an IRE-binding conformation that simultaneously enhances TfR expression while suppressing both ferritin synthesis and HIF-2α translation through 5′UTR IRE interactions. This dual mechanism restricts erythropoietic expansion when iron availability is insufficient, despite elevated erythropoietin (EPO) levels. Conversely, iron-replete states promote IRP1’s conversion to a cytosolic aconitase (holo-IRP1), relieving translational repression of HIF-2α and enabling synergistic activation of erythroid programs through HIF-2α-mediated EPO receptor sensitization and enhanced iron utilization.

**FIGURE 4:**
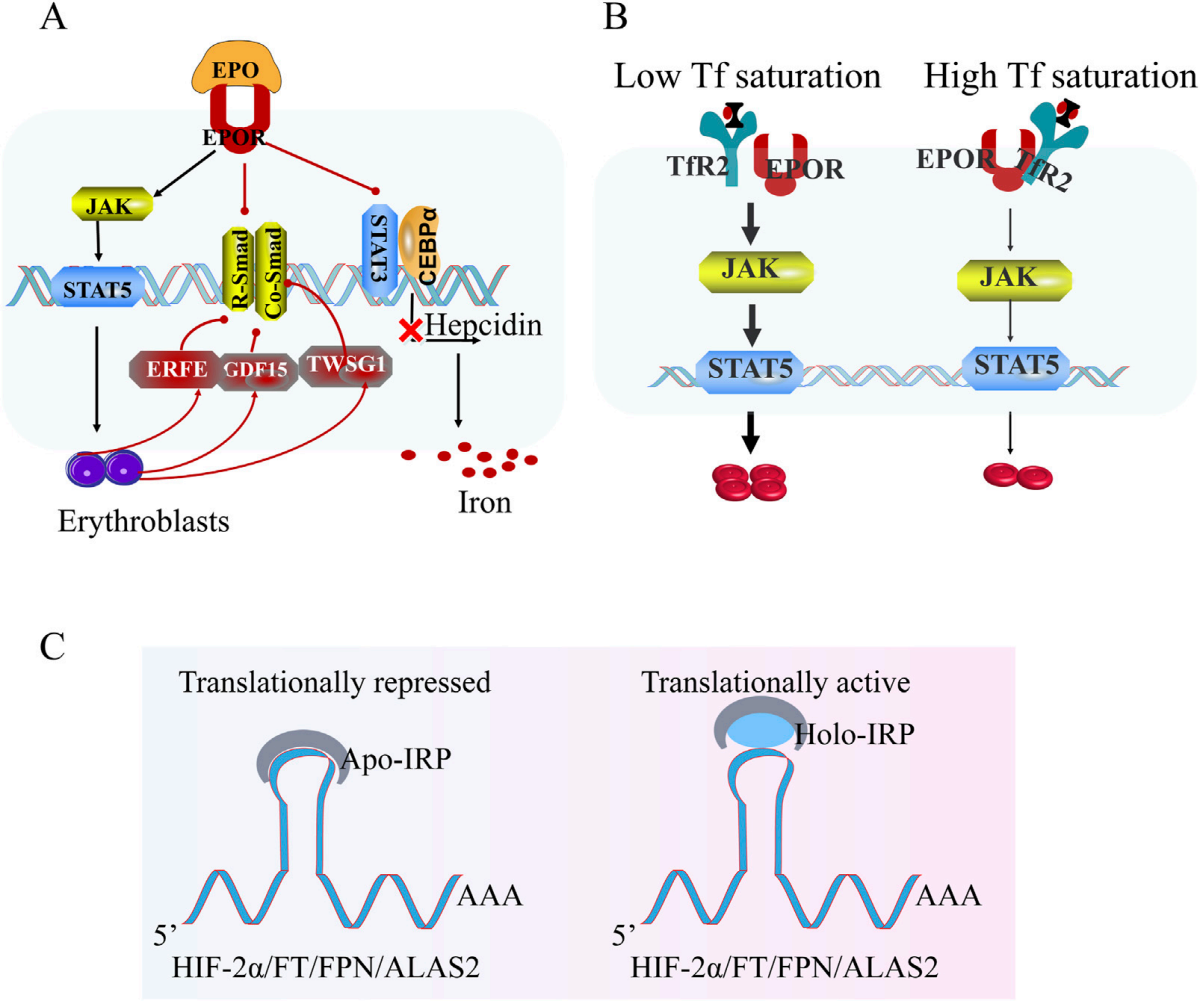
Hypoxia-regulated iron homeostasis and erythropoiesis. **(A)** Under hypoxic conditions, hypoxia-inducible factor (HIF) suppresses hepcidin transcription via inhibition of the R-Smad/Co-Smad and STAT3 signaling pathway, thereby enhancing dietary iron uptake to meet systemic demands. Erythroblast-derived factors (ERFE, GDF15, and TWSG1) further inhibit hepcidin expression, facilitating iron mobilization for hemoglobin synthesis in nascent erythrocytes. **(B)** Transferrin receptor 2 (TFR2) senses iron-saturated transferrin levels to regulate EPOR signaling dynamics. Elevated iron-saturated transferrin induces TFR2-dependent attenuation of EPOR sensitivity, suppressing downstream pro-erythroid proliferation pathways (e.g., JAK2/STAT5). Conversely, reduced iron-saturated transferrin alleviates TFR2-mediated EPOR inhibition, enhancing erythropoietin responsiveness. **(C)** Intracellular iron sensing is governed by iron regulatory proteins (IRPs): Iron deficiency promotes IRP binding to IREs, suppressing HIF-2α synthesis. Conversely, under iron-replete conditions, iron-bound IRPs dissociate from the 5′iron-responsive element (IRE) of HIF-2α mRNA, permitting HIF-2α translation and stabilization. These interconnected pathways maintain systemic iron equilibrium during hypoxic adaptation. Note: While this schematic highlights hypoxia-driven iron regulation, the precise iron metabolism network in HAPC remains undefined and warrants further investigation.

Complementing dietary iron absorption, macrophage-mediated iron recycling constitutes a critical secondary source. Specialized macrophages in the spleen and liver phagocytose senescent erythrocytes, catabolize hemoglobin via heme oxygenase-1, and export liberated iron through ferroportin for reutilization in erythropoiesis. This physiological iron cycle operates under IRP/IRE control, with hypoxia further modulating macrophage iron release through HIF-dependent regulation of ferroportin expression. The hierarchical integration of these systems—IRP-mediated post-transcriptional control, HIF-2α-driven transcriptional regulation, and macrophage iron recycling—demonstrates an evolutionarily optimized mechanism for matching erythroid demand with iron supply. Such multilayered regulation prevents pathological iron redistribution while ensuring prioritized iron allocation to erythroid precursors during hypoxic challenges (Figure 4).

The maintenance of systemic iron homeostasis represents a dynamic equilibrium governed by the interplay between iron availability and erythropoietic demand. This regulatory paradigm is exemplified by physiological adaptations to acute high-altitude hypoxia (∼4,559 m), where rapid erythropoietic activation creates transient iron-deficient conditions. Clinical observations demonstrate that short-term hypoxic exposure triggers a characteristic biomarker profile: marked elevation of soluble transferrin receptor (sTfR) and total transferrin (Tf) levels concurrent with precipitous declines in serum ferritin and transferrin saturation (TfSat) (Robach et al., 2007; Goetze et al., 2013). Notably, pre-acclimatization iron supplementation at sea level emerges as a strategic intervention to mitigate these hypobaric hypoxia-induced alterations. Prophylactic iron administration has been shown to attenuate the decline in TfSat while concurrently improving physiological adaptation metrics—including enhanced peripheral oxygen saturation (SpO2) and reduced pulmonary artery systolic pressure (PASP) (Holdsworth et al., 2020; Patrician et al., 2022).

The current understanding of iron metabolism under chronic high-altitude exposure reveals significant heterogeneity across studies, reflecting complex interactions between genetic, environmental, and pathophysiological variables. Investigations into iron status in populations with EE demonstrate inconsistent patterns. Andean residents with EE at 3,825 m exhibited no statistically different iron (serum iron, ferritin, transferrin) to non-EE (De Ferrari et al., 2014), while EE subjects at 5,100 m showed conserved systemic iron markers but elevated ERFE levels (Cairo et al., 2023). Conversely, Tibetan HAPC patients at 3,850–4,200 m displayed iron overload signatures characterized by elevated serum iron, ferritin, and transferrin (Liu et al., 2018), suggesting potential ethnic-specific regulatory divergences in iron homeostasis adaptation.

This apparent contradiction may arise from multiple factors. Firstly, Tissue-specific iron partitioning. Altitude-induced redistribution rather than systemic iron dysregulation. Adipose tissue iron accumulation coexists with decreased aortic iron content and elevated plasma non-transferrin bound iron (NTBI) in high-altitude-adapted ovine models (Liu et al., 2022a; Knutson, 2023), indicating compartmentalized iron handling. Secondly, diagnostic limitations. Current clinical biomarkers fail to capture tissue-specific iron status or pathological NTBI levels, while WHO hemoglobin adjustment criteria (0.2 g/dL increase per 1,000 m elevation) (Gonzales et al., 2018; Ocas-Córdova et al., 2018) inadequately address altitude-modified iron requirements for augmented erythropoiesis. Thirdly, therapeutic paradox. Although observational studies associate HAPC with iron overload, causal relationships remain unproven. Iron removal improves HAPC(Liu et al., 2018) and HIF-2α/iron pathway inhibition reduces EE (Zhou et al., 2023). Notably, Paul Robach’s work challenges the connection of iron and EE, demonstrating preserved iron homeostasis in EE populations despite marked erythrocytosis (Cairo et al., 2023).

This discrepancy underscores critical knowledge gaps. 1) Whether observed iron overload in HAPC represents adaptive physiology or incipient pathology. 2) How tissue-specific iron distribution impacts organ dysfunction in CMS. 3) The threshold at which altitude-induced iron accumulation triggers pathogenic consequences. These unresolved questions highlight the urgent need for systematic, multi-tissue assessments combining advanced iron quantification techniques (e.g., MRI-based liver iron content, NTBI measurements) with molecular characterization of iron regulatory networks. Such studies must account for ethnic-specific genetic adaptations in high-altitude populations while differentiating between compensatory iron mobilization and true pathological overload. Clarifying these mechanisms will inform targeted interventions—whether iron supplementation for deficient states or precision iron chelation in overload scenarios—to optimize adaptation outcomes in chronic hypoxia.

## 5 Mature erythrocyte-derived feedback circuits in HAPC pathogenesis

The progression from physiological adaptation to pathological HAPC may be driven by a dysregulated feedback loop between mature erythrocytes and erythropoietic activity. Under normal physiological conditions, circulating RBCs exert homeostatic control over erythropoiesis through oxygen-dependent regulatory mechanisms. When functional RBC mass achieves sufficient oxygen delivery, molecular signals—mediated by oxygen-sensing pathways (e.g., HIF degradation) and erythrocyte-derived factors such as ERFE—suppress EPO production and inhibit erythroid progenitor proliferation, thereby maintaining hematological equilibrium. In HAPC, however, this feedback system becomes maladaptive. Despite elevated RBC counts, dysfunctional erythrocytes—characterized by impaired deformability, reduced oxygen-release capacity, or oxidative damage—fail to generate inhibitory signals sufficient to attenuate erythropoietic drive. This creates a self-perpetuating cycle: defective RBCs inadequately resolve tissue hypoxia, sustaining EPO synthesis and promoting pathological erythroid expansion. Three interrelated mechanisms may underpin this aberrant feedback: functional impairment of RBCs, proteomic and metabolomic signaling dysregulation and apoptotic resistance/delayed clearance.

### 5.1 Functional impairment of RBCs

The functional integrity of circulating erythrocytes serves as a critical interface between erythropoietic activity and systemic oxygen delivery in HAPC. However, mechanistic investigations of RBC pathophysiology in HAPC remain constrained by inherent challenges: unlike nucleated bone marrow cells, mature RBCs lack organelles and cannot be cultured *in vitro*, limiting studies to phenotypic analyses of circulating populations. Current understanding of RBC dysfunction in HAPC thus centers on two primary axes—oxygen transport efficiency and hemodynamic contributions to blood viscosity—though critical knowledge gaps persist in linking these functional impairments to disease progression.

#### 5.1.1 Oxygen transport dynamics

The cardinal function of RBCs, oxygen carriage and release, is governed by hemoglobin-oxygen affinity (P50), deformability, and redox homeostasis. Acute hypoxic exposure triggers adaptive metabolic shifts, notably increased 2,3-bisphosphoglycerate (2,3-BPG) synthesis, which reduces hemoglobin-oxygen affinity (right-shifted oxygen dissociation curve) to enhance tissue oxygen offloading (Liu et al., 2016). Paradoxically, chronic hypoxia appears to induce contrasting adaptations. Comparative studies demonstrate that long-term Han plateau residents exhibit significantly lower P50 values (left-shifted curves) compared to lowland populations (Li et al., 2018). But not all of these residents were diagnosed with HAPC, as their [Hb] ranged from 124 to 240 g/L, with a median of 172 g/L. Studies comparing the oxygen affinity of RBCs between adaptive altitude Sherpas and Caucasians indicate that Sherpas had higher oxygen affinity (Morpurgo et al., 1976). Current evidence suggests a potential inverse relationship between hemoglobin concentration and oxygen affinity, where individuals with hemoglobin levels below 180 g/L appear better adapted to chronic hypoxia through optimized oxygen loading at pulmonary capillaries (Reeves and Leon-Velarde, 2004). However, the precise mechanistic interplay between hemoglobin concentration, oxygen affinity, and altitude adaptation remains incompletely characterized.

#### 5.1.2 Blood viscosity

The secondary rheological functions of erythrocytes critically regulate hemodynamic viscosity through four interdependent determinants: Hct, plasma viscosity, RBC deformability, and cellular aggregation. In CMS, pathologically elevated Hct (>65%) becomes the principal driver of viscous resistance, with clinical evidence establishing hyperviscosity as a primary pathogenic factor. Although elevated Hct directly increases blood viscosity, partial compensatory mechanisms attenuate this effect through physiological adaptations.

#### 5.1.3 RBC deformability

RBC deformability - governed by surface area-to-volume ratio, membrane elasticity, and cytoskeletal architecture - demonstrates hypoxia-responsive modulation. Hypoxic conditions induce deoxygenated hemoglobin binding to ankyrin, destabilizing ankyrin-Band 3 protein bridges and triggering membrane remodeling that enhances cellular flexibility (Faivre et al., 2020; Chu et al., 2016). These molecular adaptations enable erythrocytes to dynamically respond to viscosity alterations (Salvi et al., 2023), though notably, blood viscosity remains predominantly determined by Hct and plasma viscosity rather than RBC deformability or aggregation kinetics (Stauffer et al., 2020). Recent investigations revealed compromised storage stability in CMS-derived erythrocytes (HAPC subtype), potentially linked to tyrosine-phosphorylated Band 3 membrane protein modifications (Wu et al., 2023), suggesting potential quality issues in these erythrocytes.

Transgenic murine models overexpressing EPO (Hct 80%–90%) demonstrated enhanced erythrocyte flexibility through analogous adaptation mechanisms (Vogel et al., 2003). Intriguingly, comparative analyses of CMS patients and healthy high-altitude residents (5,100 m) revealed no significant differences in cellular mechanical properties or rheological parameters (Stauffer et al., 2020). This functional equivalence suggests potential clinical utility of CMS-derived erythrocytes for transfusion purposes, but provided rigorous quality assessments are implemented (Stauffer et al., 2024). However, current evaluations remain incomplete, having focused exclusively on physical cell properties without addressing critical biochemical parameters such as oxidative stress markers, oxygen dissociation kinetics, or metabolic competency. Certainly, due to frequently exchanges material with outside, functions of RBC are much more than what we think. A thorough evaluation of their components is essential, including cytoplasmic proteins, membrane proteins, metabolites, and external vesicles.

### 5.2 Proteomic and metabolomic signaling dysregulation

#### 5.2.1 Proteomic alterations

Emerging research aims to systematically link the comprehensive erythrocyte proteome to its functional repertoire. While acute hypoxia-induced proteomic changes in red blood cells (RBCs) have been extensively characterized (Jin et al., 2024), investigations into chronic hypoxia adaptation remain limited. A rodent study demonstrated that salidroside administration ameliorated membrane proteome dysregulation in HAPC models, identifying eight differentially expressed proteins associated with oxidative stress mitigation, redox homeostasis, and peroxisomal pathways (Shou-Ning et al., 2020). However, the lack of comparative analysis with non-HAPC controls limits mechanistic interpretation.

Plasma-RBC crosstalk significantly influences erythrocyte functionality. Comparative plasma proteomics revealed HAPC-associated dysregulation of complement components (C4A, C6 ↓; MASP1 ↑) and inflammatory mediators (CALR ↓; CNDP1 ↑), implicating aberrant activation of complement cascades, coagulation pathways, and immune responses (Wang et al., 2019). Elevated pro-inflammatory cytokines (IL-1β, IL-2, IL-3, TNF-α, MCP-1, IL-16) in Han Chinese HAPC populations further support systemic inflammation as a hallmark of this pathology (Yi et al., 2021; Li et al., 2024b). Notably, erythrocytes actively participate in immune regulation through complement component recycling and inflammatory mediator exchange (Hertz et al., 2023).

Multi-group proteomic comparisons (sea-level Han, high-altitude Han, HAPC patients, Tibetans) identified HAPC-specific upregulation of hemoglobin β-chain (Hb-β), thioredoxin-1 (TRX1), and phosphoglycerate kinase 1 (PGK1), suggesting dysregulated hydrogen peroxide metabolism, ROS handling, and inflammatory signaling. Resveratrol intervention attenuated HAPC progression by modulating coagulation-inflammation networks via 14-3-3 protein epsilon (up), von Willebrand factor (down), and Antithrombin-III (down), while enhancing Rho-GTPase signaling (Deng et al., 2020). These findings underscore RBCs as active modulators of inflammatory and hemostatic pathways through protein absorption/secretion mechanisms.

#### 5.2.2 Metabolic Reprogramming

Hypoxia drives extensive remodeling of erythrocyte metabolic networks. Hypoxia accelerates the remodeling of RBC metabolism, affecting pathways such as glycolysis, pentose phosphate pathway (PPP), glutathione metabolism, nitrogen metabolism, arginine and sulfur metabolism, and carboxylic acid metabolism, etc. alters (Jin et al., 2024; D'Alessandro et al., 2016). Notably, chronic hypoxia in HAPC elicits distinct metabolic profiles compared to acute hypoxia responses. Peripheral erythrocytes serve dual roles as both biomarkers of bone marrow hematopoietic activity and functional modulators of hematopoiesis through metabolic feedback mechanisms.

S1P, a hypoxia-sensitive bioactive lipid enriched in mature erythrocytes, demonstrates altitude-responsive dynamics. In controlled hypoxic exposure (5,260 m, 16 days), healthy lowlanders exhibited progressive erythrocyte S1P accumulation correlating with elevated sphingosine kinase 1 (Sphk1) activity and enhanced hemoglobin oxygen release (Sun et al., 2016). Murine models revealed Sphk1-driven S1P elevation protects against acute hypoxic tissue damage, though its role in chronic hypoxia remains unverified. Notably, erythrocyte longevity-associated metabolites—including adenosine, S1P, and glutathione-related amino acids—were identified as potential biomarkers of hypoxic adaptation (Yu et al., 2025), positioning oxygen-dependent metabolic regulation as a therapeutic target.

Peripheral RBC metabolism directly influences bone marrow function through regulatory networks. Arginine supplementation alleviates CMS in rats via microRNA-144-5p-mediated pathways (Zhang et al., 2023). Genetic loci associated with RBC distribution width (RDW) variants regulate metabolic homeostasis (Feng et al., 2023).

#### 5.2.3 Extracellular vesicle-mediated pathological signaling

Plasma-derived extracellular vesicles (EVs) from Andean HAPC patients exacerbate endothelial inflammation and oxidative stress in HUVEC models. Tibetan HAPC cohorts exhibit distinct exosomal miRNA profiles featuring upregulated: hsa-miR-122-5p, hsa-miR-423-5p, hsa-miR-4433b-3p, hsa-miR-1291, and hsa-miR-106b-5p (Wang et al., 2023). Concurrently, elevated miR-451a and miR-210-3p accelerate CD34^+^ progenitor erythroid differentiation (Liu et al., 2023), while erythrocyte-derived miRNAs (e.g., miR-451a) associated with cardiovascular events may indirectly worsen systemic hypoxia through tissue-damage pathways (Kontidou et al., 2023).

### 5.3 Apoptotic resistance and delayed clearance

Polycythemia may arise from prolonged erythrocyte survival or attenuated cell death. Circulating erythrocytes exhibit species-specific lifespans: 100–120 days in humans, 35–50 days in mice, and 40–55 days in rats (Belcher and Harriss, 1959). Apoptotic clearance (eryptosis) involves phosphatidylserine (PS) externalization, cell shrinkage, membrane blebbing, and macrophage-mediated phagocytosis. Notably, a 1954 study reported comparable erythrocyte lifespans in Andean HAPC patients (109–117 days) and healthy controls (Berlin et al., 1954), though modern validation of these findings is lacking.

In HAPC rodent models, erythrocytes display reduced eryptotic stimuli, including diminished cytosolic Ca^2+^ levels and annexin-V binding, alongside elevated CD47 expression—a “do not eat me” signal inhibiting macrophage phagocytosis (Tang et al., 2019). Paradoxically, despite suppressed eryptosis, these models exhibit increased osmotic fragility and morphological abnormalities, suggesting dysfunction in macrophage phagocytosis.

Single-cell RNA sequencing (scRNA-seq) reveals significant depletion of splenic red pulp macrophages in HAPC. Because, reduced Ccl2, Ccl7, and Csf1 expression impairing monocyte recruitment to splenic niches. Hypoxia-induced ferroptosis via Nuclear Receptor Coactivator 4 (NCOA4)-mediated ferritin degradation may further modulate erythrocyte turnover. Iron release through autolysosomal NCOA4 activity fuels Fenton reactions, promoting lipid peroxidation (Yang et al., 2024). However, existing studies focus on short-term hypoxia (≤14 days, hemoglobin ≤210 g/L), leaving chronic HAPC-associated ferroptosis mechanisms unresolved.

Current literature bifurcates into: phenotypic descriptions of HAPC eryptosis without mechanistic exploration; generalized eryptosis mechanisms not contextualized to HAPC pathophysiology. Rigorous and comprehensive studies are needed to thoroughly investigate the mechanisms underlying HAPC pathophysiology.

## 6 Conclusion

Hypoxia-driven [Hb] elevation serves as a compensatory mechanism for oxygen transport but progresses to pathological conditions such as HAPC during decompensation phases. Maintaining [Hb] levels within an altitude-optimized range—elevated above sea-level baselines yet below pathological thresholds—is critical for sustaining physiological oxygen delivery while avoiding hemodynamic dysfunction. This review synthesizes systemic pathophysiological alterations across the erythrocyte lifecycle in HAPC: Diagnostic Limitations: Current [Hb]-centric diagnostic criteria inadequately distinguish adaptive erythrocytosis from HAPC pathology, risking both overdiagnosis in resilient populations and in underdiagnosis vulnerable groups. Erythropoietic Equilibrium: Physiological erythrocyte homeostasis relies on a dynamic balance between proliferation (driven by SENP1, EPO, HIF2α, lncRNA HIKER) and apoptosis (ARID1B). HAPC disrupts this equilibrium, favoring EE. Microenvironmental Regulation: Emerging evidence implicates hematopoietic niche dysregulation and iron metabolism in HAPC pathogenesis, though mechanistic insights remain preliminary.

**FIGURE 5:**
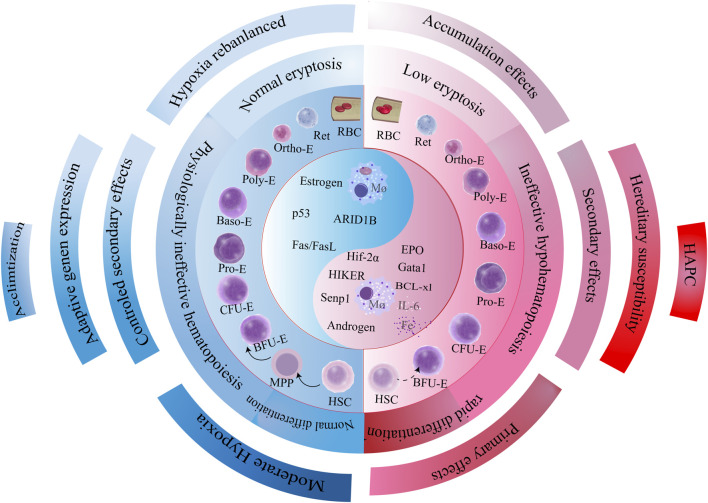
Pathogenetic cascade of HAPC. Genetic predisposition governs the hypoxic response trajectory, eliciting three divergent downstream pathways that collectively drive pathologic erythrocyte population dynamics. This cascade culminates in imbalanced erythropoiesis characterized by accelerated erythroid production and attenuated apoptosis, with hyperproliferation serving as the core driver of erythrocytosis. Mechanistically, bone marrow erythroid precursors exhibit dysregulated proliferation-apoptosis equilibrium, mediated through synergistic activation of pro-survival signaling (e.g., EPO/HIF axis) and suppression of apoptotic executors (e.g., BCL-2 family modulation). The self-reinforcing cycle of erythroid expansion establishes the pathologic foundation of HAPC progression.

We propose three interconnected hypotheses to guide future therapeutic interventions (Figure 5).

### 6.1 Hypothesis I: primary hypoxic drivers

#### 6.1.1 Multifactorial hypoxia

Pathological erythrocytosis arises from compounded hypoxic stressors, including sleep-related hypoxemia and perinatal hypoxia (Perger et al., 2022), a, exacerbated by cardiopulmonary-renal maladaptation leading to critically low oxygen saturation (Pham et al., 2017). This aligns with Leon-Velarde’s model of altitude decompensation (Villafuerte et al., 2022).

#### 6.1.2 Dysfunctional erythropoiesis

Hypoxia-induced erythrocytes exhibit impaired oxygen transport capacity, paradoxically stimulating compensatory overproduction. HAPC should be redefined as a pan-erythrocytic pathology involving cellular dysfunction across the lifecycle, rather than a binary [Hb] threshold. Morphological abnormalities (e.g., acanthocytes) may initiate pathological feedback loops, though mechanistic links require validation.

### 6.2 Hypothesis II: secondary pathogenic amplifiers

Primary hypoxia-triggered erythrocytosis is compounded by:

#### 6.2.1 Oxidative stress and inflammation

Disrupted hematopoietic feedback via ROS accumulation and cytokine dysregulation (IL-1β, TNF-α) (Yi et al., 2021; Li et al., 2024b).

#### 6.2.2 Metabolic derangements

Hypoxia-altered glycolysis, glutathione cycling, and arginine metabolism (Jin et al., 2024).

### 6.3 Hypothesis III: cumulative altitude exposure

We postulate a pathophysiological continuum hypothesis wherein non-indigenous migrants lacking the evolutionary-acquired genetic adaptations characteristic of high-altitude native populations demonstrate inevitable progression to CMS when subjected to sustained residency at critical altitude thresholds. This disease trajectory appears governed by two cardinal determinants: 1) exposure duration exhibiting positive correlation relationship with hematological maladaptation severity, and 2) altitude-dependent potentiation of cumulative hypoxic injury.

## 7 Therapeutic strategies and perspectives

Current therapeutic strategies for HAPC involved four principal domains, each with distinct mechanisms and limitations.

Therapeutic phlebotomy (Therapeutic erythrocyte apheresis) remains a cornerstone intervention for rapid hematocrit reduction, temporarily improving tissue oxygenation by hemodilution (Ouyang et al., 2024; Dong et al., 2020). However, its transient efficacy is underscored by reactive erythrocytosis recurrence within weeks, necessitating repeated procedures (Yan et al., 2025). Emerging evidence suggests that adjunctive Traditional Chinese Medicine may partially mitigate this limitation, potentially through pleiotropic mechanisms requiring further pharmacological validation (Rivera-Ch et al., 2007).

Ventilatory modulation strategies have demonstrated variable success. Pharmacological agents such as acetazolamide—a carbonic anhydrase inhibitor—exhibit sustained benefits by enhancing hypoxic ventilatory responses (Richalet et al., 2005; Sharma et al., 2017; Richalet et al., 2008). Similarly, dopaminergic antagonists, medroxyprogesterone, and almitrine show efficacy in improving respiratory drive (Sharma et al., 2017).

Traditional Chinese Medicine, notably Tibetan medicine, offer multi-target effects by attenuating oxidative stress, modulating inflammatory cascades, and improving microcirculation (Liu et al., 2022b; Chen et al., 2021; Deng et al., 2020). Despite promising phenotypic outcomes, their therapeutic specificity remains poorly defined, highlighting the need to identify precise molecular targets.

Lifestyle modifications: Lifestyle intervention is an excellent preventive measure. Epidemiological studies implicate dietary factors—tsampa as a risk amplifier versus ghee tea’s protective effects—in HAPC progression, likely mediated through lipid metabolism (Cui et al., 2022). Exercise-induced benefits may arise from dual mechanisms: hemolysis-mediated erythrocyte turnover and enhanced pulmonary gas exchange efficiency [155].

Critically, interventions targeting erythropoietic regulation remain underdeveloped. Preclinical studies on HIF-2α inhibitors (e.g., PT2385) show promise in suppressing erythrocytosis (Li et al., 2024a). Hematopoiesis is tightly regulated, thus requiring cautious intervention. We suggest three strategic axes emerge for future therapeutic innovation. HIF-EPO pathway disruption: Combined blockade of HIF transcriptional activity and iron regulatory protein (IRP) signaling to break their synergistic amplification loop. Hematopoietic microenvironments interventions: Antioxidant and anti-inflammatory targeting within hematopoietic microenvironments, building upon traditional medicine paradigms but advancing to cytokine/spatial transcriptomic resolution.

Metabolic Reprogramming: Elucidating erythroid metabolic plasticity (e.g., glycolysis-to-oxidative phosphorylation shifts) during hypoxic adaptation—a virtually unexplored frontier in HAPC treatment.

Despite growing clinical recognition of HAPC, the lack of comprehensive mechanistic elucidation has severely constrained the development of effective therapies. We propose that unraveling the molecular and cellular underpinnings of HAPC should be a critical focus in subsequent research endeavors, paving the way for mechanism-driven therapeutic innovations.
